# Quaternized Molecular Brush‐Grafted Injectable Microgel with Anti‐Inflammatory and Drainage Properties for Efficient Therapy of Anal Fistula

**DOI:** 10.1002/advs.202407537

**Published:** 2024-12-09

**Authors:** Runxian Wang, Pengwei Ma, Siqi He, Xiao Wang, Jinquan Zhang, Junwen Ye, Mingli Su, Xingxing Shi, Ruoxu Dou

**Affiliations:** ^1^ The Fifth Affiliated Hospital Sun Yat‐sen University Zhuhai 519000 P. R. China; ^2^ School of Chemistry Sun Yat‐sen University Guangzhou 510006 P. R. China; ^3^ Department of General Surgery (Colorectal Surgery) Guangdong Institute of Gastroenterology Guangdong Provincial Key Laboratory of Colorectal and Pelvic Floor Diseases The Sixth Affiliated Hospital Sun Yat‐sen University Guangzhou 510655 P. R. China; ^4^ Department of General Surgery (Endoscopic Surgery) Guangdong Institute of Gastroenterology Guangdong Provincial Key Laboratory of Colorectal and Pelvic Floor Diseases The Sixth Affiliated Hospital Sun Yat‐sen University Guangzhou 510655 P. R. China; ^5^ The Eighth Affiliated Hospital Sun Yat‐sen University Shenzhen 518033 P. R. China

**Keywords:** anal fistula, anti‐inflammatory, carbon nanotube, injectable microgel, quaternized molecular brush

## Abstract

Anal fistula is a common disease with recurrent inflammation and accumulated exudate. Traditional treatments often fail to effectively eliminate inflammation and ensure adequate drainage, leading to prolonged healing time and a high recurrence rate. Herein, a new class of quaternized molecular brush‐grafted injectable microgel (denoted as GAA@CNT‐*g*‐PVBTMA) is developed through thermal polymerization and mechanical fragmentation to promote the healing process of anal fistula. Benefiting from the fragmented morphology with a porous structure, the microgel can effectively fill the fistula and facilitate the drainage of exudate. Owing to the electrostatic interactions between the positively charged quaternized carbon nanotube molecular brush (CNT‐*g*‐PVBTMA) and the negatively charged inflammatory cytokines, GAA@CNT‐*g*‐PVBTMA microgel exhibits excellent anti‐inflammatory properties with scavenging rates of 92.6% for tumor necrosis factor‐α (TNF‐α) and 92.5% for interleukin‐1β (IL‐1β). Rat inflammatory anal fistula model demonstrates that the microgel can effectively reduce inflammation and epithelialization of the fistula, thereby promoting the healing of the anal fistula. By integrating effective filling, adequate drainage, and excellent anti‐inflammatory properties, GAA@CNT‐*g*‐PVBTMA microgel provides a promising new direction for the treatment of anal fistula.

## Graphical abstract

A new class of quaternized molecular brush‐grafted injectable microgel is developed through thermal polymerization and mechanical fragmentation, which not only achieves effective filling of the fistula and facilitates the drainage of exudate, but also exhibits excellent anti‐inflammatory property by capturing negatively charged inflammatory cytokines, thereby effectively promoting healing of anal fistula.

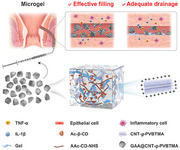

## 1 Introduction

Anal fistula is a common anorectal disease characterized by the presence of an abnormal tract connecting the anal canal/rectum to perianal skin,^[^ ^1^ ^]^ leading to persistent inflammation and an increased susceptibility to malignancy.^[^ ^2^ ^]^ Surgery is typically the primary treatment by removing the fistulous tract and ensuring drainage.^[^ ^3^ ^]^ However, complex anal fistula often requires extensive surgical dissection,^[^ ^4^ ^]^ which may lead to prolonged healing and catastrophic incontinence,^[^ ^5^ ^]^ with a recurrence rate up to 57% and incontinence rate up to 52%.^[^ ^6^ ^]^ Therefore, it is critical to develop an effective and meanwhile less traumatic therapeutic strategy for anal fistula.

**Figure 1:**
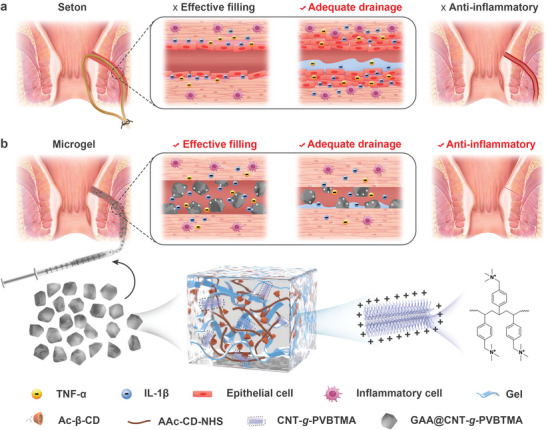
Schematic diagram of anti‐inflammatory and drainage properties of GAA@CNT‐*g*‐PVBTMA microgel in the treatment of anal fistula. a) After anal seton surgery, the seton is placed in the fistula to facilitate the drainage of exudate. However, the seton cannot provide effective physical filling to exclude inflammatory stimuli from the fistula, and constant stimuli will cause abnormal epithelialization of the fistula wall and hinder the healing process. b) Our GAA@CNT‐*g*‐PVBTMA microgel can capture negatively charged inflammatory cytokines through the electrostatic interactions between CNT‐*g*‐PVBTMA and inflammatory cytokines, and integrate effective filling, adequate drainage, and anti‐inflammatory properties to promote healing of anal fistula. [Correction added on 2 January 2025, after first online publication: figure 1 is replaced with the updated version.]

Less invasive therapeutic options for anal fistula currently include seton, bioprosthetic plug, and fibrin glue. A seton is placed in the fistula to facilitate the drainage of exudate without causing incontinence.^[^ ^7^ ^]^ However, the seton cannot provide an effective physical filling to exclude inflammatory stimuli from the fistula, and constant stimuli will cause abnormal epithelialization of the fistula wall and hinder the healing process (**Figure** **1**a).^[^ ^8^ ^]^ Meanwhile, seton therapy typically requires a prolonged period for effective drainage and gradual healing, which may lead to a lengthy treatment process and cause long‐term discomfort for patients. Alternatively, materials such as bioprosthetic plug^[^ ^9^ ^]^ and fibrin glue^[^ ^10^ ^]^ have been used to fill the fistula without extensive surgery.^[^ ^11^ ^]^ Bioprosthetic plug is typically made from porcine intestinal submucosa or human dermal matrix with superior biocompatibility.^[^ ^12^ ^]^ Similarly, fibrin glue is composed of fibrinogen and thrombin and therefore biologically safe.^[^ ^13^ ^]^ However, these materials will block the drainage after filling the fistula^[^ ^14^ ^]^ with a healing rate of less than 50%.^[^ ^15^ ^]^ Therefore, the balance between effective filling and adequate drainage is crucial for anal fistula treatment. Moreover, the persistent inflammation and abnormal epithelialization of fistula are featured by local accumulation of the inflammatory cytokines, such as tumor necrosis factor‐α (TNF‐α) and interleukin‐1β (IL‐1β).^[^ ^16^ ^]^ Currently, there is no effective clinical therapy to neutralize inflammatory cytokines. Thus, a biocompatible and anti‐inflammatory material that is simultaneously filling and draining is highly desirable for the treatment of anal fistula.

Herein, a new class of quaternized molecular brush‐grafted injectable microgel (denoted as GAA@CNT‐*g*‐PVBTMA) with excellent anti‐inflammatory and drainage properties is successfully developed by thermal polymerization of quaternized carbon nanotube molecular brush (CNT‐*g*‐PVBTMA) with precursor solution and mechanical fragmentation (Figure 1b). The precursor solution is mainly composed of gelatin (Gel), acrylic acid N‐succinimidyl ester (AAc‐NHS), and acrylated β‐cyclodextrin (Ac‐β‐CD). Due to the incorporation of CNT‐*g*‐PVBTMA, our GAA@CNT‐*g*‐PVBTMA microgel exhibits a low swell ratio of 9.9%, which could maintain stable mechanical property in the wet environment. Owing to the fragmented morphology with a porous structure, our microgel demonstrates good fluidity during injection and forms a stable seal once the shear forces are removed, which can achieve effective filling of the fistula and facilitate the drainage of exudate. More importantly, our GAA@CNT‐*g*‐PVBTMA microgel can achieve high scavenging rates of 92.6% for TNF‐α and 92.5% for IL‐1β relying on the electrostatic interactions between the positively charged CNT‐*g*‐PVBTMA and the negatively charged inflammatory cytokines. The rat inflammatory anal fistula model confirms that our microgel can reduce inflammation and epithelialization of anal fistula, and effectively promote fistula healing. Therefore, our work provides a new effective strategy for the treatment of anal fistula.

## 2 Results and Discussion

**Figure 2:**
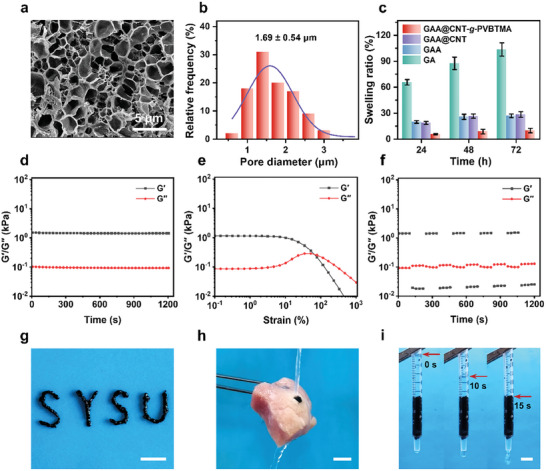
Characterization and performance evaluation of GAA@CNT‐*g*‐PVBTMA microgel. a) SEM image of freeze‐dried GAA@CNT‐*g*‐PVBTMA microgel. b) The pore size distribution of freeze‐dried GAA@CNT‐*g*‐PVBTMA microgel. c) Swelling ratios of GA, GAA, GAA@CNT, and GAA@CNT‐*g*‐PVBTMA microgels in PBS solution for 72 h at 37 °C (*n* = 3). d–f) Oscillation time scanning (d), strain amplitude scanning (e), and cyclic amplitude scanning (f) of GAA@CNT‐*g*‐PVBTMA microgel. g) Digital photo showing good injectability of GAA@CNT‐*g*‐PVBTMA microgel to form the letters “SYSU” (scale bar: 1 cm). h) Digital photo of GAA@CNT‐*g*‐PVBTMA microgel firmly adhering to a 4‐mm‐diameter through hole in the porcine tissue under water flushing (scale bar: 1 cm). i) Digital photo showing good filling and draining properties of GAA@CNT‐*g*‐PVBTMA microgel in PBS solution (scale bar: 5 mm).

The preparation of our GAA@CNT‐*g*‐PVBTMA microgel includes the synthesis of the quaternized molecular brush (i.e., CNT‐*g*‐PVBTMA), thermal polymerization of a precursor solution composed of Gel, AAc‐NHS, and Ac‐β‐CD, and mechanical fragmentation. GAA@CNT microgel was prepared following the same steps as GAA@CNT‐*g*‐PVBTMA microgel, except for replacing CNT‐*g*‐PVBTMA with carbon nanotube (CNT). Similarly, GA and GAA microgels were prepared from the given precursor solutions of Gel/AAc‐NHS and Gel/AAc‐NHS/Ac‐β‐CD, respectively. CNT‐*g*‐PVBTMA was synthesized by grafting poly[(ar‐vinylbenzyl)trimethylammonium] (PVBTMA) brushes from CNT, which was characterized by X‐ray photoelectron spectroscopy (XPS) and thermogravimetric (TGA). The presence of N 1s and Cl 2p peaks in XPS spectra (Figure S1, Supporting Information) and the increased weight loss in TGA curves (Figure S2, Supporting Information) indicate the successful graft of PVBTMA. SEM image in Figure S3 (Supporting Information) displays inherited 1D morphology of CNT with a roughened surface after grafting CNT‐*g*‐PVBTMA. Moreover, the macroscopic particle size and microscopic morphology of the microgel were characterized. After mechanical fragmentation using silicone tubing, the digital photo and particle size of GAA@CNT‐*g*‐PVBTMA microgel are shown in Figure S4 (Supporting Information), indicating an average particle diameter of 1.51 mm. As shown in **Figure** **2**a,b, the freeze‐dried GAA@CNT‐*g*‐PVBTMA microgel exhibits a microporous structure with an average pore diameter of 1.69 µm. In contrast, GAA microgel possesses macroporous structure (Figure S5, Supporting Information). These results demonstrate the successful fabrication of GAA@CNT‐*g*‐PVBTMA microgel.

The anti‐swelling ability and injectability of microgels are crucial for the treatment of anal fistula.^[^ ^17^ ^]^ As shown in Figure 2c, GA microgel exhibits a swelling ratio of 103.6% after soaking in PBS solution for 72 h. In comparison, GAA and GAA@CNT microgels show significantly lower swelling ratios of 26.9% and 28.5%, respectively, due to the increased cross‐linked network structure between Ac‐β‐CD and Gel/AAc‐NHS. After the incorporation of CNT‐*g*‐PVBTMA, GAA/CNT‐*g*‐PVBTMA microgel demonstrates excellent anti‐swelling property with a low swelling ratio of 9.9% (Figure 2c). Rheological tests were conducted to analyze the moduli changes of GAA@CNT‐*g*‐PVBTMA microgel. At room temperature (25 °C), the storage modulus (G′) of GAA@CNT‐*g*‐PVBTMA microgel is always higher than the loss modulus (G″) over time, suggesting a stable gel network of the microgel (Figure 2d). The strain amplitude scanning curves of GAA@CNT‐*g*‐PVBTMA microgel reveal that the intersection of G′ and G″occurs at a shear strain of 70% (Figure 2e). As the shear strain increases beyond this point, G' becomes lower than G″and the gel network is destroyed. The modulus changes of the microgel were further assessed through cyclic amplitude scanning. As depicted in Figure 2f, G' exceeds G″ at low strain (1%) and indicates its gel state; G′ decreases significantly at high strain (300%), suggesting the breakdown of gel network structure.^[^ ^18^ ^]^ When the strain changes to low strain (1%) again, G′ can quickly return to the initial state. As shown in Figure S6 (Supporting Information), GAA@CNT‐*g*‐PVBTMA microgel can pass smoothly through irregularly shaped silicone tubing and exhibit good injectability (Video S1, Supporting Information). Moreover, our GAA@CNT‐*g*‐PVBTMA microgel can be injected to form the letters “SYSU” and retain its solid gel state once the shear forces are removed (Figure 2g). These results demonstrate that our GAA@CNT‐*g*‐PVBTMA microgel exhibits excellent anti‐swelling ability and injectability.

In clinical practice, achieving the optimal balance between effective filling and adequate drainage is essential for the successful treatment of anal fistula.^[^ ^1^ ^]^ Excessive filling may hinder proper drainage, while insufficient filling may expose the fistula to constant inflammatory stimuli and delay the healing process. As shown in Figure S7 (Supporting Information), a 4‐mm‐diameter through hole was created in the porcine tissue. Our GAA@CNT‐*g*‐PVBTMA microgel can effectively fill the entire hole and exhibit firm adhesion under water flushing (Figure 2h; Video S2, Supporting Information). To further evaluate the adhesion property of the microgel to the anal fistula tissue, we established a rat inflammatory anal fistula model by inserting a 1.6‐mm‐diameter wire through the perianal and anal area of the rat. After 14 days, the anal fistula tissue was harvested for adhesion characterization. Our GAA@CNT‐*g*‐PVBTMA microgel also demonstrates strong adhesion to the anal fistula tissue under water flushing (Video S3, Supporting Information). When the microgels are placed in a syringe, GAA@CNT‐*g*‐PVBTMA microgel can not only effectively fill the syringe, but also promote the drainage of PBS solution, which is attributed to the fragmented morphology with a porous structure (Figure 2i; Video S4, Supporting Information). Even after soaking in PBS solution for 3 days, our microgel still maintains excellent drainage property (Video S5, Supporting Information). These results demonstrate that our GAA@CNT‐*g*‐PVBTMA microgel exhibits both effective filling and draining properties, making it a superior material for the treatment of anal fistula.

**Figure 3:**
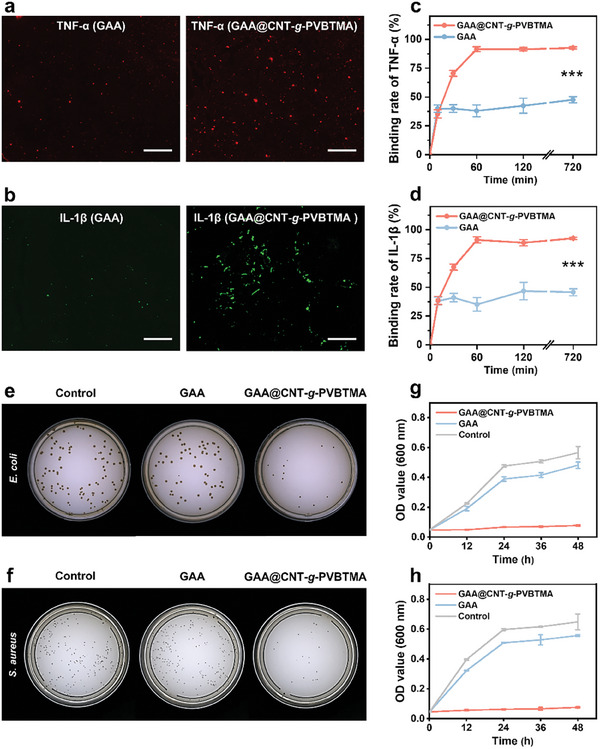
In vitro anti‐inflammatory and antibacterial properties of GAA and GAA@CNT‐*g*‐PVBTMA microgels. a,b) Immunofluorescence staining images of TNF‐α (a) and IL‐1β (b) captured by GAA and GAA@CNT‐*g*‐PVBTMA microgels (scale bars: 200 µm). c,d) Binding rates of TNF‐α (c) and IL‐1β (d) for GAA and GAA@CNT‐*g*‐PVBTMA microgels. e,f) Images of bacterial colonies of *E. coli* (e) and *S. aureus* (f) for 12 h. g,h) Optical density of *E. coli* (g) and *S. aureus* (h) suspensions for the control, GAA, and GAA@CNT‐*g*‐PVBTMA groups for 48 h. The data are presented as mean ± SD (*n* = 3; ^***^ *p* < 0.001).

The accumulation of inflammatory cytokines and bacterial infections in anal fistula disrupts normal tissue repair mechanisms and can severely delay the healing process.^[^ ^19^ ^]^ Thus, effective anti‐inflammatory and antibacterial therapies are crucial for accelerating the healing of anal fistula. To assess the anti‐inflammatory properties of GAA and GAA@CNT‐*g*‐PVBTMA microgels, their abilities to capture the negatively charged inflammatory cytokines (e.g., TNF‐α and IL‐1β) were investigated using rat anal fistula tissue homogenate to simulate inflammatory environment. GAA and GAA@CNT‐*g*‐PVBTMA microgels were incubated in the homogenate for 720 min, and immunofluorescence staining images of TNF‐α and IL‐1β captured by the microgels were taken using a fluorescence microscope. As shown in **Figure** **3**a,b, GAA@CNT‐*g*‐PVBTMA microgel shows a higher binding affinity for TNF‐α and IL‐1β compared to GAA microgel, suggesting its higher efficiency in capturing inflammatory cytokines. The binding capacities of the microgels for the inflammatory cytokines are further quantitatively investigated. GAA microgel scavenges 47.5% of TNF‐α and 45.7% of IL‐1β after 720 min of incubation. In contrast, GAA@CNT‐*g*‐PVBTMA microgel achieves significantly higher binding efficiency, scavenging 92.6% of TNF‐α and 92.5% of IL‐1β, respectively (Figure 3c,d). The incorporation of positively charged molecular brushes in the microgel may capture more negatively charged inflammatory cytokines through electrostatic interactions, thereby improving scavenging efficiency.^[^ ^20^ ^]^ The antibacterial properties of the microgels against *E. coli* and *S. aureus* were evaluated using the agar plate incubation method, as displayed in Figure 3e,f. The GAA@CNT‐*g*‐PVBTMA group shows fewer colony‐forming unit (CFU) of *E. coli* and *S. aureus* compared to the control and GAA groups. Additionally, GAA and GAA@CNT‐*g*‐PVBTMA microgels were co‐cultured with *E. coli* and *S. aureus* for 48 h, and the optical density (OD) values of the bacterial suspensions were measured to evaluate the antibacterial efficacy. As shown in Figure 3g,h, the OD values of *E. coli* and *S. aureus* suspensions in the control and GAA groups exhibit a significant increase and suggest a rapid bacterial growth phase. In contrast, the GAA@CNT‐*g*‐PVBTMA group maintains a significantly lower OD value for 48 h, demonstrating that our GAA@CNT‐*g*‐PVBTMA microgel can effectively inhibit bacterial proliferation over a long period of time. These results demonstrate that our GAA@CNT‐*g*‐PVBTMA microgel exhibits excellent anti‐inflammatory property and good bacteriostatic property by capturing inflammatory cytokines and preventing bacteria growth.

**Figure 4:**
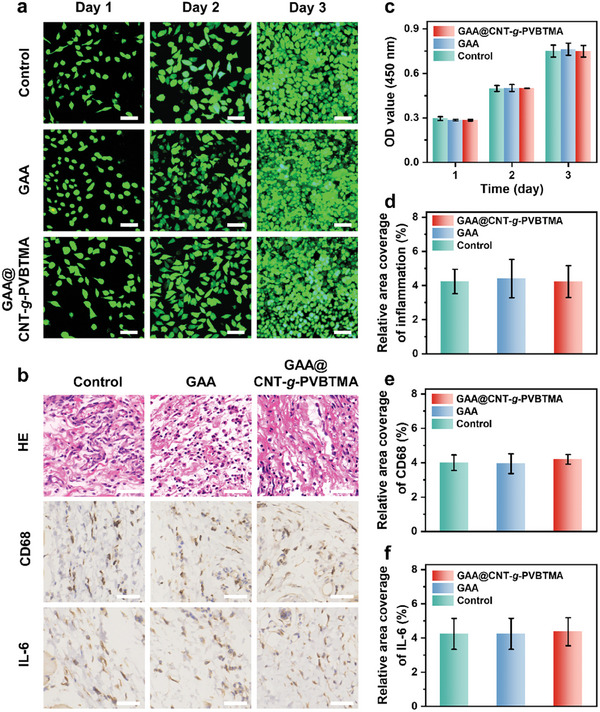
In vitro and in vivo biocompatibility of GAA and GAA@CNT‐*g*‐PVBTMA microgels. a) In vitro fluorescence images of L929 fibroblasts cultured after 1, 2, and 3 days in a control media (DMEM), GAA microgel media, and GAA@CNT‐*g*‐PVBTMA microgel media, respectively (scale bars: 100 µm). b) In vivo images of HE staining and immunohistochemical staining of CD68 and IL‐6 for the control, GAA, and GAA@CNT‐*g*‐PVBTMA groups after rat subcutaneous implantation for 3 days (scale bars: 50 µm). c) CCK‐8 assay of L929 fibroblasts cultured with GAA and GAA@CNT‐*g*‐PVBTMA microgels after 1, 2, and 3 days. d–f) Quantitative analysis of inflammatory cells (d), CD68 (e), and IL‐6 (f).

Good biocompatibility is crucial for injectable implant materials.^[^ ^21^ ^]^ The biocompatibility of GAA and GAA@CNT‐*g*‐PVBTMA microgels was assessed using live/dead cell viability assay and CCK‐8 assay. As shown in **Figure** **4**a, the morphology and cell density of L929 fibroblasts in the GAA and GAA@CNT‐*g*‐PVBTMA groups are comparable to those of the control group. Meanwhile, the quantitative analysis of CCK‐8 assay reveals no significant statistical differences in proliferation among the groups, demonstrating good in vitro biocompatibility of GAA and GAA@CNT‐*g*‐PVBTMA microgels (Figure 4c). To further evaluate in vivo biocompatibility, subcutaneous injections of 0.9% saline (serving as control), GAA microgel, and GAA@CNT‐*g*‐PVBTMA microgel were administered into the dorsal skin of rats, respectively. Tissue samples from the injection sites after 3 days were excised and subjected to HE and immunohistochemical staining to assess inflammatory responses, as shown in Figure 4b. The HE staining results show no statistical differences in the infiltration areas of inflammatory cells (Figure 4d). Moreover, immunohistochemical staining was conducted to investigate two inflammatory markers, including CD68 (a macrophage marker) and IL‐6 (an inflammatory cytokine).^[^ ^22^ ^]^ As shown in Figure 4e,f, there are no statistical differences in the expressions of CD68 and IL‐6 among all the groups. These results demonstrate the excellent biocompatibility of GAA and GAA@CNT‐*g*‐PVBTMA microgels both in vitro and in vivo.

**Figure 5:**
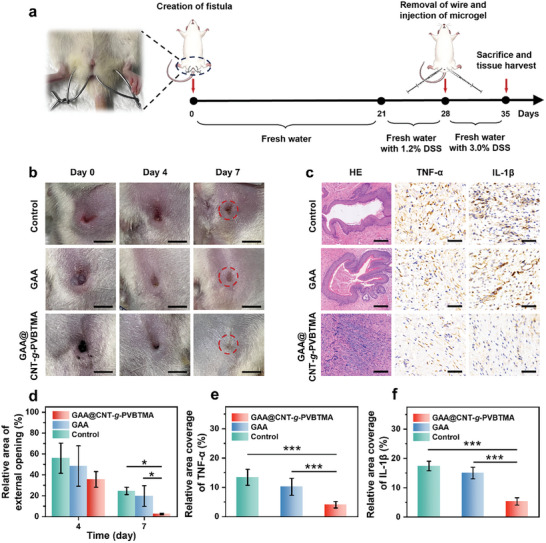
Evaluation of in vivo healing efficacy of GAA and GAA@CNT‐*g*‐PVBTMA microgels in rat inflammatory anal fistula model. a) Schematic diagram of the establishment of rat inflammatory anal fistula model. b) Digital photos of anal fistula wounds after treatment with GAA or GAA@CNT‐*g*‐PVBTMA microgels (scale bars: 5 mm). c) Images of HE staining and immunohistochemical staining of TNF‐α and IL‐1β for the control, GAA, and GAA@CNT‐*g*‐PVBTMA groups (scale bars: 250 µm in column 1, 50 µm in columns 2 and 3). d) Relative area of external opening in rat inflammatory anal fistula model. e,f) Quantitative analysis of TNF‐α (e) and IL‐1β (f). The data are presented as mean ± SD (*n* = 5; ^*^ *p* < 0.05, ***p < 0.001).

To further evaluate in vivo healing efficacy of GAA@CNT‐g‐PVBTMA microgel, a rat inflammatory anal fistula model was induced by inserting a thicker wire through the perianal and anal area and giving fresh drinking water with dextran sodium sulfate (DSS).^[^ ^23^ ^]^ After leaving the wire in place for 28 days, the tissue sample from one of the rats was subjected to HE staining to evaluate the establishment of the animal model (Figure 5a). The HE staining images clearly show a fistula with complete epithelialization, indicating the successful establishment of the anal fistula model (Figure S8, Supporting Information).^[^ ^24^ ^]^ As shown in **Figure 5b**, the healing status of the fistula external opening in different groups is observed after 7 days. The external opening of the GAA@CNT‐*g*‐PVBTMA group is almost completely closed with a relative external opening area of 3%. In comparison, the control group exhibits significantly slower healing, with 25% of the relative area remaining unhealed (Figure 5d). Rats were then euthanized on day 7, and the fistula tissues were harvested for histology analysis including HE staining and immunohistochemical staining of TNF‐α and IL‐1β. The GAA@CNT‐*g*‐PVBTMA group shows dense fibroproliferative scars, while the control and GAA groups exhibit the presence of fistula with epithelialized cells (Figure 5c). Quantitative analysis reveals that the expressions of TNF‐α and IL‐1β are significantly lower in the GAA@CNT‐*g*‐PVBTMA group compared to the other two groups (Figures 5e,f). To further evaluate the degree of in vivo inflammation, the fresh anal fistula tissue homogenate was taken from rats after 7 days of treatment. For comparison, homogenates from healthy perianal tissue were collected as the normal group. ELISA tests were conducted to measure the amount of IL‐1β and TNF‐α in the anal fistula tissue homogenate. The GAA@CNT‐*g*‐PVBTMA group exhibits lower levels of inflammatory cytokines compared to the control group. Although cytokine levels in the GAA@CNT‐*g*‐PVBTMA group remain slightly elevated compared to the normal group, there are no statistically significant differences in the levels of inflammatory cytokines between the two groups (Figure S9, Supporting Information), indicating that GAA@CNT‐*g*‐PVBTMA microgel exhibits excellent anti‐inflammatory property by significantly reducing the levels of the negatively charged inflammatory cytokines. To further evaluate the role of GAA@CNT‐*g*‐PVBTMA microgel in inducing M2 macrophages polarization in vivo, we conducted immunohistochemical analysis for two markers: iNOS, a marker for pro‐inflammatory M1 macrophages, and CD206, a marker for anti‐inflammatory M2 macrophages. As shown in Figure S10 (Supporting Information), the GAA@CNT‐*g*‐PVBTMA group shows a lower expression of iNOS and higher expression of CD206 compared to the control and GAA groups. These results show that our GAA@CNT‐*g*‐PVBTMA microgel can effectively induce the polarization of M2 macrophages. By integrating excellent anti‐inflammatory and drainage properties, our GAA@CNT‐*g*‐PVBTMA microgel can effectively reduce inflammation and epithelialization of the fistula, thereby promoting the healing of anal fistula.

## 3 Conclusion

In conclusion, we have successfully developed a new class of quaternized molecular brush‐grafted injectable microgel through thermal polymerization and mechanical fragmentation. Benefitting from the incorporation of CNT‐*g*‐PVBTMA, our GAA@CNT‐*g*‐PVBTMA microgel exhibits excellent anti‐swelling property with a low value of 9.9%. Due to the fragmented morphology with a porous structure, our GAA@CNT‐*g*‐PVBTMA microgel can effectively fill the fistula and facilitate the drainage of exudate. Remarkably, our microgel can capture inflammatory cytokines of the fistula through electrostatic interactions and exhibit excellent anti‐inflammatory property, achieving remarkable scavenging rates of 92.6% for TNF‐α and 92.5% for IL‐1β. The rat inflammatory anal fistula model confirms that our microgel can effectively reduce inflammation and epithelialization of the fistula, and promote the fistula healing. Thus, our work provides a promising and effective therapy for the treatment of anal fistula.

## 4 Experimental Section

The Experimental Section is available in the Supporting Information.

## Conflict of Interest

The authors declare no conflict of interest.

## Supporting information



Supporting Information

Supplementary Video 1

Supplementary Video 2

Supplementary Video 3

Supplementary Video 4

Supplementary Video 5

## Data Availability

The data that support the findings of this study are available from the corresponding author upon reasonable request.
