# Supplementary material for: Quaternized Molecular Brush‐Grafted Injectable Microgel with Anti‐Inflammatory and Drainage Properties for Efficient Therapy of Anal Fistula
Source: Adv Sci (Weinh). 2024 Dec 9;12(5):2407537. doi: 10.1002/advs.202407537 (PMC11791944; doi:10.1002/advs.202407537)
Supplement: Supplementary file 1 — Supporting Information [file ADVS-12-2407537-s001.docx]

**Supporting Information**

**Quaternized Molecular Brush-grafted Injectable Microgel with Anti-inflammatory and Drainage Properties for Efficient** **Therapy of Anal Fistula**

*Runxian Wang, Pengwei Ma, Siqi He, Xiao Wang, Jinquan Zhang, Junwen Ye*, Mingli Su*, Xingxing Shi*, Ruoxu Dou**

R. Wang, S. He, X. Wang, J. Zhang, R. Dou

The Fifth Affiliated Hospital

Sun Yat-sen University

Zhuhai 519000, P. R. China

P. Ma

School of Chemistry

Sun Yat-sen University

Guangzhou 510006, P. R. China

X. Shi

The Eighth Affiliated Hospital

Sun Yat-sen University

Shenzhen 518033, P. R. China

J. Ye

Department of General Surgery (Colorectal Surgery)

Guangdong Institute of Gastroenterology

Guangdong Provincial Key Laboratory of Colorectal and Pelvic Floor Diseases

The Sixth Affiliated Hospital

Sun Yat-sen University

Guangzhou 510655, P. R. China

M. Su

Department of General Surgery (Endoscopic Surgery)

Guangdong Institute of Gastroenterology

Guangdong Provincial Key Laboratory of Colorectal and Pelvic Floor Diseases

The Sixth Affiliated Hospital

Sun Yat-sen University

Guangzhou 510655, P. R. China

*E-mails:

dourx@mail.sysu.edu.cn; shixx7@mail.sysu.edu.cn;

suml5@mail.sysu.edu.cn; yejw23@mail.sysu.edu.cn

# Experimental Section

## Materials

Gelatin (Gel), β-cyclodextrin (β-CD, 98%), triethylamine (TEA, 99%), ammonium persulfate (APS), vinylbenzyl trimethylammonium chloride (VBTMA, 97%), N,N,N′,N′′,N′′-pentamethyldiethylenetriamine (PMDETA, 99%), copper (I) bromide (CuBr, >99%) and dimethylformamide (DMF, 99.8%) were purchased from Shanghai Aladdin Biochemical Technology Co., Ltd. (China). Carbon nanotube (CNT) was purchased from Shanghai Macklin Biochemical Co., Ltd. (China). Acrylic acid N-succinimidyl ester (AAc-NHS, 98%) was purchased from Anhui Zesheng Technology Co., Ltd. (China). Phosphate buffer saline (PBS) was purchased from Wuhan Servicebio Technology Co., Ltd. (China). Dextran sulfate sodium (DSS) was purchased from Dalian Meilun Biotechnology Co., Ltd. (China). Cell counting kit-8 (CCK-8) and calcein AM/PI double staining kit were purchased from Beijing Solarbio Science & Technology Co., Ltd. (China). Standard fibroblast cell line L929 fibroblasts were provided from the Cell Bank of the Chinese Academy of Sciences (China). Staphylococcus aureus (*S. aureus*, ATCC 6538) and Escherichia coli (*E. coli*, ATCC 25922) were obtained from Guangdong Microbial Culture Collection Center (China). The female Sprague Dawley (SD) rats (220-250 g) were provided from Animal Center of Sun Yat-sen University (China). The enzyme linked immunosorbent assay (ELISA) kits were purchased from Wuhan Fine Biotech Co., Ltd. (China). All solvents and chemicals were purchased from commercial sources and used without further purification.

## Synthesis of CNT-*g*-PVBTMA

Br-modified CNT (CNT-Br) was prepared by the typical method described previously.^[1]^ Poly[(ar-vinylbenzyl)trimethylammonium]-grafted CNT (CNT-*g*-PVBTMA) was prepared by the following method. Typically, 200 mg of CNT-Br, 3.2 g of VBTMA, 63 μL of PMDETA, and 20 mL of DMF were added into a 50 mL Schlenk flask to obtain a homogeneous suspension after ultrasonic treatment for 0.5 h. The mixture was further stirred for 0.5 h under N_2_ atmosphere. Subsequently, 43 mg of CuBr was added into the mixture with bubbling N_2_ for anot­­her 0.5 h. Finally, the Schlenk flask was placed in an oil bath at 75 °C for 12 h. After stepwise treatments of diluting with DMF, filtrating, washing, and freeze-drying, CNT-*g*-PVBTMA was obtained.

## Synthesis of Ac-β-CD

Ac-β-CD was prepared by the typical method described in a previous work.^[2]^ Typically, 10 g of β-CD was added into 150 mL of DMF, followed by the addition of 7 mL of TEA. The mixture was stirred and cooled down to 0 °C before 5 mL of acrylic acid was added into the solution. After stirring for 12 h, the mixture was dripped into 200 mL of acetone to precipitate the modified β-CD. The precipitate was washed several times with acetone and vacuum dried to obtain Ac-β-CD.

## Preparation of microgels

GAA@CNT-*g*-PVBTMA microgel was prepared as follows: First, 0.8 g of gelatin was dissolved in 7.5 mL of deionized water with 0.4 g of AAc-NHS and 0.4 g of Ac-β-CD added into the solution. Then, 100 mg of CNT-*g*-PVBTMA and 54 mg of APS were added into the mixture, with each addition followed by stirring and sonicating for 3-5 min. Finally, the mixture was heated in a blast oven at 60 °C for 45 min, washed with deionized water and crushed using silicone tubing with a 1.2-mm-inner-diameter to obtain GAA@CNT-*g*-PVBTMA microgel.

GAA@CNT microgel was prepared following the same steps as the GAA@CNT-*g*-PVBTMA microgel, except for replacing 100 mg of CNT-*g*-PVBTMA with 100 mg of CNT.

GAA microgel was prepared following the same steps as the GAA@CNT-*g*-PVBTMA microgel, except for the incorporation of 100 mg of CNT-*g*-PVBTMA.

GA microgel was prepared as follows: First, 0.8 g of gelatin was dissolved in 7.5 mL of deionized water with 0.4 g of AAc-NHS added into the solution. Then, 54 mg of APS were added into the mixture, with each addition followed by stirring and sonicating for 3-5 min. Finally, the mixture was heated in a blast oven at 60 °C for 45 min, washed with deionized water and crushed using silicone tubing with a 1.2-mm-inner-diameter to obtain GA microgel.

## Material characterization

The microstructures were investigated by a field emission scanning electron microscope (FE-SEM, Hitachi S-4800). Before examination, all samples were freeze-dried and sprayed with platinum. The pore sizes in the SEM image were analyzed using Image J, where the maximum pore diameter of 100 pores was randomly measured. Thermogravimetric (TGA) analysis was characterized with a thermogravimetric analyzer (TGA, Netzsch TGA209F1), with the temperature increased to 900 °C at a rate of 10 °C/min under N_2_ atmosphere. X-ray photoelectron spectroscopy (XPS, Thermo-VG Scientific ESCALAB 250Xi) with a standard Al Ka X-ray source was used to analyze the chemical structure. Rheological tests were measured using the advanced rotational rheometer (TA Discovery HR-20). Swelling ratios of microgels were measured at 37 °C. Samples were immersed in a PBS solution at different times, and then taken out to wipe off the PBS in the surface of samples using filter paper before measuring weight. The swelling ratio is calculated as follows:

$$swelling ratio=\frac{W-W_{0}}{W_{0}} \times100\%$$

where $W$ is the weight of microgels after predetermined time in the PBS solution, and $W_{0}$ represents the beginning weight.

## Cytokines binding test

GAA and GAA@CNT-*g*-PVBTMA microgels were incubated in 10 mL of rat anal fistula tissue homogenate for 12 h at 37 °C and 5% CO_2_. The anal fistula tissue homogenate was prepared by collecting fresh anal fistula tissue from rats, which was then ground with 1 mL of PBS per 0.1 g of tissue at 4 °C until adequately homogenized. After incubation, samples of the supernatant were centrifuged and collected at intervals of 10, 30, 60, 120, and 720 min, and the concentrations of inflammatory cytokines (i.e., TNF-α and IL-1β) were quantified using ELISA kits. The binding rates of the microgels were evaluated by analyzing the cytokine concentrations in the homogenate before and after incubation. For cytokine binding analysis, a portion of microgels from the above experiment were washed thoroughly with PBS solution and sent to Wuhan Servicebio Technology Co., Ltd. for immunofluorescence staining.

## Antibacterial assessment

The antibacterial property against *E. coli* and *S. aureus* of GAA and GAA@CNT-*g*-PVBTMA microgels was evaluated by counting the number of colony-forming unit (CFU) on broth agar plates. Prior to bacterial inoculation, microgels were exposed to UV light for 12 h. Colonies of *E. coli* and *S. aureus* were cultured in Luria-Bertani (LB) liquid medium and recovered by continuous shaking at 37 ℃ and 5% CO_2_ for 12 h. Briefly, 1g of GAA or GAA@CNT-*g*-PVBTMA microgels were immersed in 10 mL of *E. coli* or *S. aureus* suspension (10^6^ CFU mL^−1^), followed by incubation at 37 °C for 12 h. The supernatants of each sample were diluted 10^6^-fold with PBS, and 100 μL of the diluted bacterial suspension was seeded on broth agar plate to assess the efficacy of preventing bacteria growth. Additionally, the optical density (OD) value of the bacterial suspension was measured at 600 nm.

## In vitro biocompatibility

GAA and GAA@CNT-*g*-PVBTMA microgels were sterilized by UV irradiation for 1 h and then immersed in a culture medium (microgel content: 100 mg mL^-1^) for 12 h to obtain microgel extracts. L929 fibroblasts were seeded at a density of 8×10^3^ cells per well in 96-well plates and cultured in a humidified incubator at 37 °C with 5% CO_2_. As a control, the cells were seeded in the culture medium containing 100 μL of DMEM supplemented with 10% fetal bovine serum and 1% penicillin-streptomycin. After 12 h of incubation, L929 fibroblasts adhered to the plates, and then the culture medium was replaced with microgel extracts. Subsequently, 100 µL of 10% CCK-8 solution was added to each well and incubated for 2 h. The OD value at 450 nm was measured using a full wavelength microplate reader to evaluate the cell viability. On the other hand, live/dead cell viability assay was performed by adding 100 μL of calcein acetoxymethyl ester (calcein-AM) solution (10 μg mL^-1^) under dark conditions into another well and incubated for 15 min. The stained L929 fibroblasts were observed under an inverted fluorescence microscope.

## In vivo biocompatibility

All animal surgery protocols were reviewed and approved by the Animal Ethics Committee of Guangzhou Boyao Biomedical Technology Co., Ltd. (IAEC-K-230517-05). Adult female SD rats (220-250 g, Sun Yat-sen University) were used for the in vivo biocompatibility tests. Prior to the procedures, GAA and GAA@CNT-*g*-PVBTMA microgels were sterilized under UV light for 2 h. All rats were anesthetized with isoflurane (1%-2% isoflurane in oxygen) in an anesthetizing chamber, and then incisions were made on their backs. The microgels were injected into subcutaneous incisions, while 0.9% saline was injected and served as control. After 3 days, the animals were euthanized by CO_2_ inhalation. The excised subcutaneous tissues were then fixed in 4% paraformaldehyde for 24 h and sent to Wuhan Servicebio Technology Co., Ltd. for HE staining and immunohistochemical staining.

## Anal fistula repair experiment in rat

To establish a rat inflammatory anal fistula model, a 1.6-mm-diameter galvanized wire was used to pierce from the anus toward the perianal skin at the 3 o’clock and 9 o’clock positions and retained for 28 days. Dextran sulfate sodium (DSS) at concentrations of 1.2% was administered continuously for one week before the removal of the wire, and a concentration of 3% was administered for one week after the removal of wire. We divided 15 rat fistulas into three groups. The first group (n=5) was left untreated as the control. The second group (n=5) was injected with GAA microgel, and the third group (n=5) was treated with GAA@CNT-*g*-PVBTMA microgel to thoroughly fill the fistula. The healing status of the external fistula openings were observed and statistically analyzed after 7 days of treatment. Finally, the rats were euthanized by CO_2_ inhalation, and the anal fistula tissues were collected for HE staining and immunohistochemical staining of TNF-α and IL-1β. The staining procedures were performed by Wuhan Servicebio Technology Co., Ltd.

## Statistical Analysis

All statistical analyses were performed using Origin software. The data were presented as mean ± standard deviation. Statistical differences between the groups were determined using one-way analysis of variance (ANOVA). Statistical significance was indicated by * for p < 0.05, ** for p < 0.01, and *** for p < 0.001.


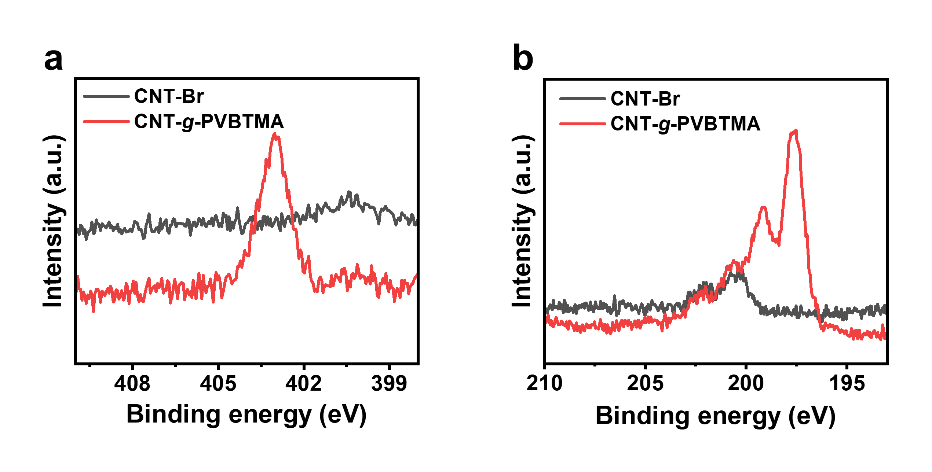


**Figure S1.** XPS spectra of (a) N 1s and (b) Cl 2p for CNT-Br and CNT-*g*-PVBTMA.


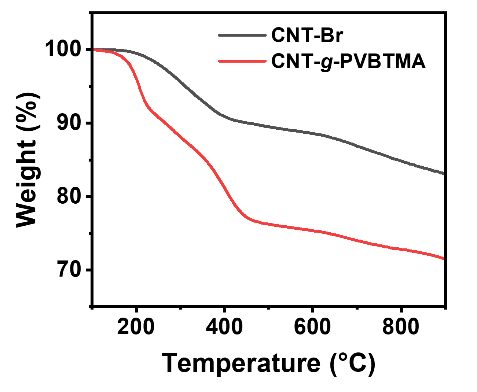


**Figure S2.** TGA curves of CNT-Br and CNT-*g*-PVBTMA under N_2_ atmosphere.


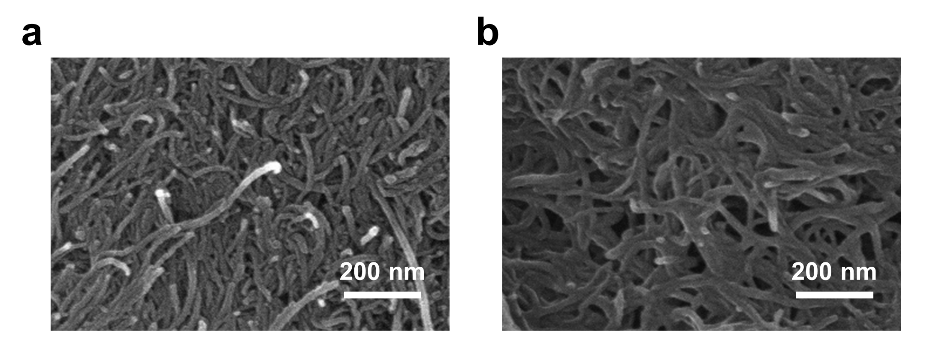


**Figure S3.** SEM images of (a) CNT-Br and (b) CNT-*g*-PVBTMA.


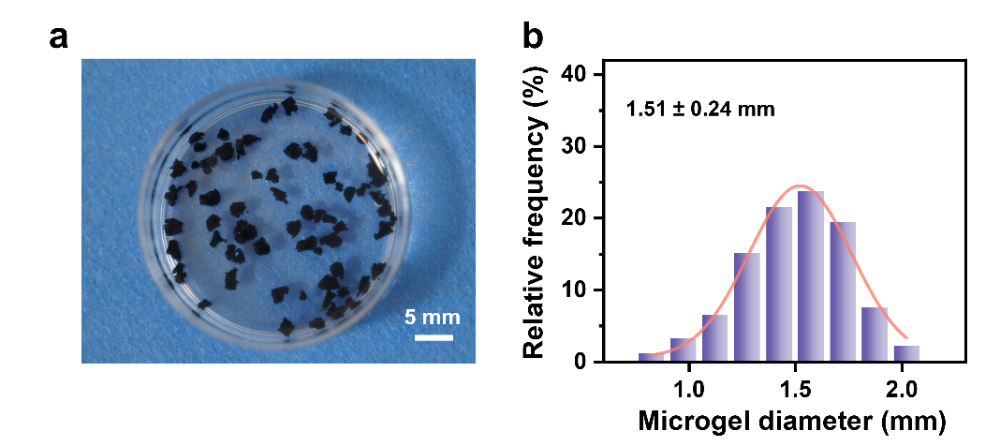


**Figure S4.** (a) Digital photo and (b) particle size distribution of GAA@CNT-*g*-PVBTMA microgel.


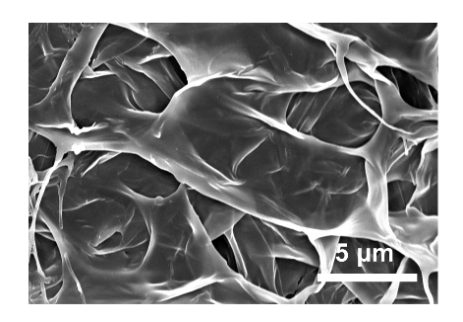


**Figure S5.** SEM image of freeze-dried GAA microgel.


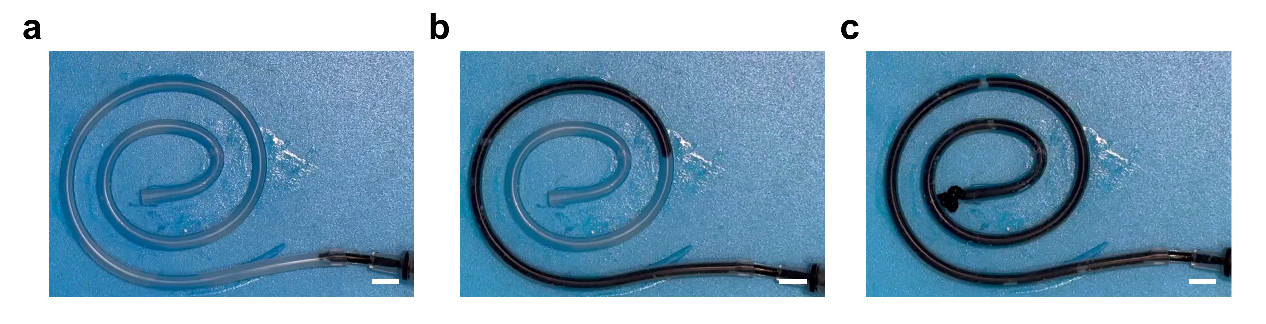


**Figure S6.** Digital photos of GAA@CNT-*g*-PVBTMA microgel in silicone tubing: (a) before injection, (b) during injection, and (c) after injection (scale bars: 5 mm).


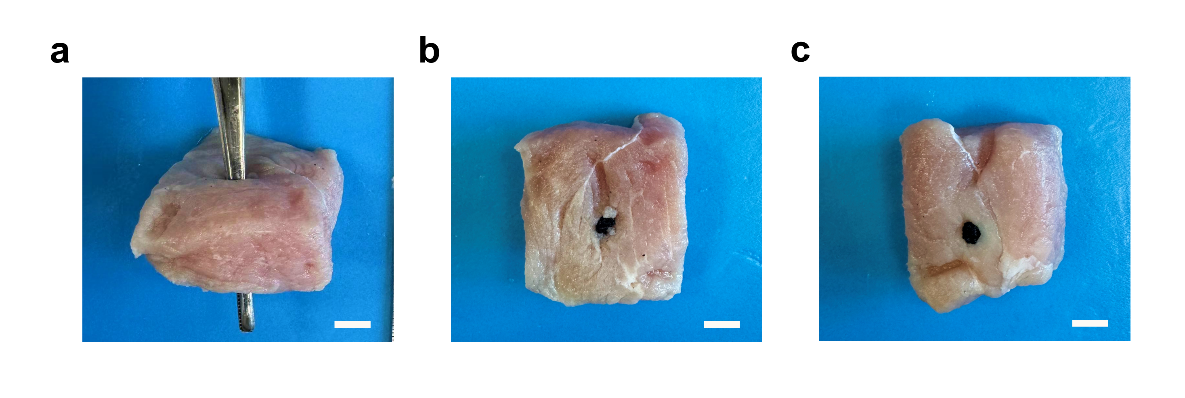


**Figure S7.** (a) Digital photo of the porcine tissue with 4 mm diameter through hole (scale bar: 1 cm). (b, c) Digital photos of top surface (b) and bottom surface (c) of the porcine tissue after filling the GAA@CNT-*g*-PVBTMA microgel (scale bars: 1 cm).


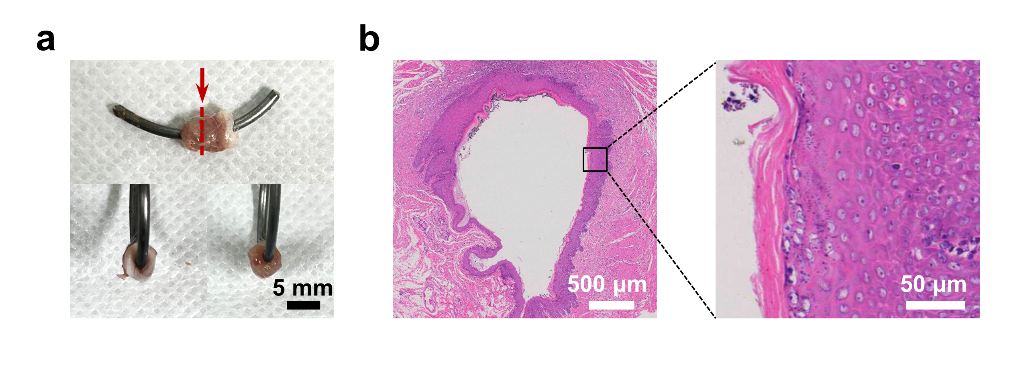


**Figure S8.** (a) Digital photo of the mature anal fistula. Tissue samples from the cross-section pointed by red arrow are excised and subjected to HE staining. (b) HE staining images of the mature anal fistula.


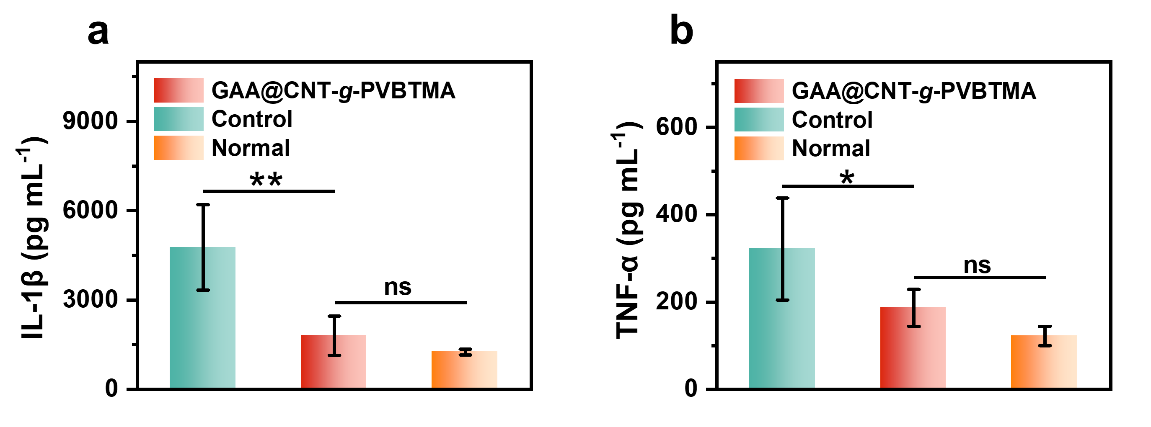


**Figure S9.** (a) IL-1β and (b) TNF-α concentrations of the anal fistula tissue homogenate for the control, normal, and GAA@CNT-*g*-PVBTMA groups. The data are presented as mean ± SD (n = 3-5; * p < 0.05, ** p < 0.01).


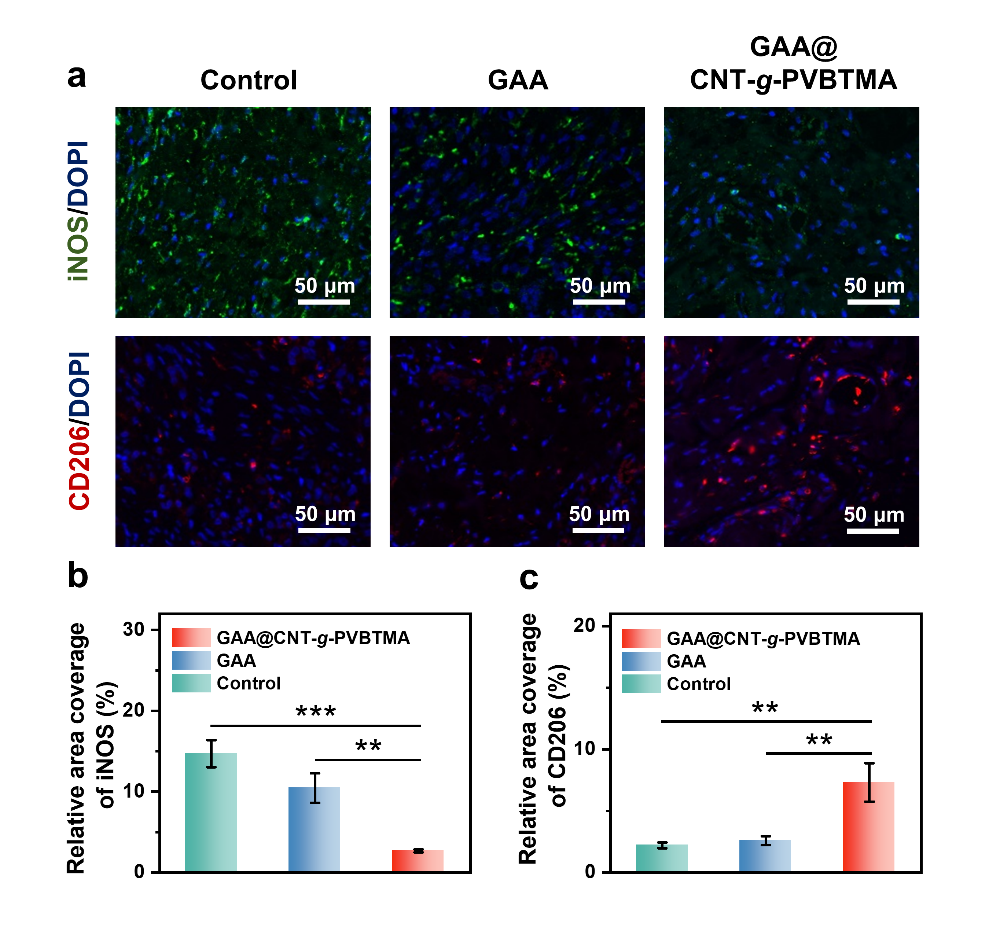


**Figure S10.** (a) Immunofluorescence staining images of iNOS and CD206 for the control, GAA and GAA@CNT-*g*-PVBTMA groups. (b, c) Quantitative analysis of iNOS-positive cells (b) and CD206-positive cells (c). The data are presented as mean ± SD (n = 3, ** p < 0.01, *** p < 0.001).

# References

[1] a) S. Srivastava, *Adv. Mater. Lett.* **2013**, *4* (1), 2; b) Y. Liang, L. Chen, D. Zhuang, H. Liu, R. Fu, M. Zhang, D. Wu, K. Matyjaszewski, *Chem. Sci.* **2017**, *8* (3), 2101.

[2] Q. Feng, K. Wei, S. Lin, Z. Xu, Y. Sun, P. Shi, G. Li, L. Bian, *Biomaterials* **2016**, *101*, 217.
